# Ultrasound protocol in odontogenic infections: a new proposal

**DOI:** 10.4317/medoral.25583

**Published:** 2022-10-16

**Authors:** Samuel Macedo Costa, Bruna Campos Ribeiro, Alessandro Oliveira de-Jesus, Gustavo Rezende Libanio, Roger Lanes-Silveira, Marcio Bruno Figueiredo Amaral

**Affiliations:** 1 Orcid: 0000-0002-6906-9407. DDS, OMFS, FBCOMS, Ph.D. Student. Oral and Maxillofacial Surgery Ph.D. Program, University of São Paulo FORP-USP, Ribeirão Preto, Brazil; 2 Orcid: 0000-0002-1430-959X. DDS, OMFS Resident. Oral and Maxillofacial Surgery Residency Program, University of São Paulo FORP-USP, Ribeirão Preto, Brazil; 3 Orcid: 0000-0003-1858-1096. DDS, MSC, OMFS, FBCOMS. Resident of the Oral and Maxillofacial Surgery Service of the João XXIII Hospital/ Hospitalar Foundation of Minas Gerais, Belo Horizonte, Brazil; 4 Orcid: 0000-0003-1983-6716. DDS, MSC, OMFS. Chief of the Oral and Maxillofacial Surgery Service of the João XXIII Hospital/ Hospitalar Foundation of Minas Gerais, Belo Horizonte, Brazil; 5 Orcid: 0000-0003-2907-8066. DDS, MD, OMFS, PhD. Professor of the Oral and Maxillofacial Surgery Service of the João XXIII Hospital/ Hospitalar Foundation of Minas Gerais, Belo Horizonte, Brazil; 6 Orcid: 0000-0003-0350-5170. DDS, OMFS, Ph.D. Head of the Oral and Maxillofacial Surgery Residency Service of the João XXIII Hospital/ Hospitalar Foundation of Minas Gerais, Belo Horizonte, Brazil

## Abstract

**Background:**

Point-of-care-ultrasound can be applied to preview a difficult airway, detect the presence of fluid collection, and soft-tissue edema, and guide the drainage location, although is rarely used. The purpose of this study is to validate a protocol for the assessment of these clinical features on patients with severe odontogenic infections.

**Material and Methods:**

This was a single-group prospective cohort study (n=20) including patients with the diagnosis of deep-neck propagation of odontogenic infection. A transcervical linear high-frequency probe transducer (13-6 MHz) was used to scan the structures of the upper airway and the infectious collections. The drainage was guided by ultrasound and the patients were daily evaluated, according to the protocol. The data were extracted and the airway volume, midline deviation, and other important data such as length of hospital stay, dysphagia, voice alteration, raised floor of the mouth, dyspnea, and neck swelling were registered.

**Results:**

The ultrasound examination was correlated with multiple clinical findings, such as dyslalia (*p*=0,069), dysphagia (*p*=0,028), dyspnea (*p*=0,001), among others. This protocol has an advantage as it can be used at bedside evaluation, allowing the assessment of severe and unsTable patients, and predicting the increase of the hospitalization time (*p*=0,019).

**Conclusions:**

This protocol is reliable for the assessment of the upper airway, even in an emergency, predicting not only the severity of the clinical features but aids in the determination of the length of the hospitalization time.

** Key words:**Ultrasound, doppler ultrasound imaging, ultrasound imaging, infection.

## Introduction

Deep-neck odontogenic infections are a relevant segment of the Oral and maxillofacial surgery daily practice, causing significant morbidity and mortality (1,2). Usually, this condition arises from the apical tooth region and vestibular spaces, disseminating to the more hazardous cervical spaces, causing severe complications such as mediastinitis, necrotizing fasciitis, sepsis, and thoracic empyema, osteomyelitis, and especially airway deviation or obstruction (3,4). Delayed treatment may result in a fast and hazardous propagation to deep neck spaces promoting significant morbidity and mortality (1-5).

An extensive literature screening provided by Moghimi (5), describes the spreading of these infections, and the most-frequent deep fascial spaces involved are the submandibular, sublingual, buccal, lateral pharyngeal, masticator, and submental. Signs and symptoms are common as fever, trismus, extra-oral facial swelling, the floor of the mouth swelling, speech and swelling disorders, dyspnea, voice alterations, tachycardia, and tachypnea are the ones more represented in the literature (6).

The appropriate management of the airways is of utmost importance and it has to consider the maximum mouth opening and the neck anatomy. The pus collections and the local edema may distort the local reference points and deviate the airway, which can further hinder conventional laryngoscopy and intubation (2).

In patients with deep neck infections related to odontogenic causes, the presence of signs of difficult intubation and extubation are clear as mouth opening limitation, excessive swelling, and the presence of infectious collections (1,2). Urgent airway intervention carries a risk of complications that can lead to severe morbidity and even death, together with those errors in the planning for the extubation of these patients usually course in airway compromise (2,6).

The management of odontogenic infections remains controversial, however, it is well established in the literature that an aggressive and surgical-oriented treatment must be preconized. The infection source should be controlled and the causative teeth may be removed. Conservative management can be recklessly leading to up 15% of failure (1,7,8).

There is enough evidence in the English-language literature to support the use of preoperative imaging to support surgical treatment, providing the anatomical spacing of the collections, and limiting surgical incision and drainage spaces (9). The contrast-enhanced computed tomography (CT SCAN) is the gold standard for the imaging of odontogenic infections, being an excellent modality for the diagnosis of potentially life-threatening infections (1,6,9).

Ultrasound is a fast, cost-effective, porTable, and radiation-free technique that has been increasingly studied in several areas and is a potential modality for the airway assessment of patients with severe odontogenic infections, with deep neck progression (1,9). The Point-of-care ultrasound (POCUS) is well-established in emergency departments worldwide, being both a diagnostic and an interventional tool for a variety of procedures. POCUS can be applied to preview a difficult airway, or subglottic stenosis, to predict the size of the endotracheal tube for pediatric patients, to confirm intubation, and even to identify the cricothyroid membrane (10). Together with that, specifically for the patient with odontogenic infections, it can detect the presence of fluid collection, soft-tissue edema, and drainage location with sensitivity and specificity comparable to the magnetic resonance image (MRI) and computed tomography (CT SCAN) (1,9).

This study provides a validation of a protocol for the assessment of severe odontogenic infections in the submandibular, sublingual, lateral pharyngeal, and upper neck regions of patients. In addition, this study evaluates the upper airway of the patients with a prospective tridimensional analysis of the upper airway space and correlation with clinical signs of airway disturbances.

A correlation between immaginological and clinical signs of severe odontogenic infection is also provided.

## Material and Methods

- Study design and patients

This was a single-group prospective cohort study of twenty consecutive patients with the diagnosis of deep-neck propagation of odontogenic infection in the Oral and Maxillofacial Surgery Service of the João XXIII Hospital (Belo Horizonte, Brazil) from July 2020 to November 2020. Patients were included if they had symptoms and signs of odontogenic infection with deep-neck propagation, confirmed by clinical and immunological examination. The exclusion criteria consisted of 1) patients below 18 years old, 2) missing data or incomplete medical records, and 3) patients who were tracheostomized, as the airway analysis could be missed or messed up. All the patients were evaluated daily by the same surgeon (S.M.C.) until discharge.

All the patients included had agreed and signed a consent informed term. This study followed all the recommendations of the Helsinki Protocol and had been approved by the Ethics Committee under the number: 92432218.2.0000.5119.

- Ultrasound protocol technique

A “Xario 100MX Ultrasound” (Toshiba, Tokyo, Japan) was used for the bedside evaluation of severe odontogenic infections on the first day of hospitalization, followed by a daily assessment until the patient's discharge. A Transcervical linear high-frequency probe transducer (13-6 MHz, Toshiba, Tokyo, Japan) was employed to scan the structures of the upper airway and the structures of the submandibular, sublingual, lateral pharyngeal, and upper cervical regions. No intra-oral probe was used for US examination, reducing the risk of presenting device bias.

First Step: Carotid Bifurcation and Submandibular Area

**Figure 1:**
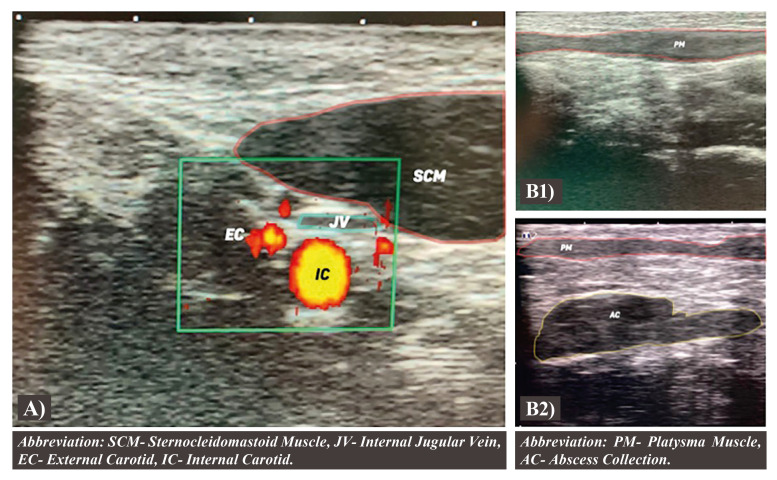
A) Ultrasonography image with doppler enhanced study of the carotid bifurcation region revealing the sternocleidomastoid, internal jugular vein, and internal and external carotid. B) Ultrasonography image of the submandibular area, left side normal and right side affected. It is necessary to assess the relationship between the platysma and the adjacent structures. On the affected side, an abscess collection is observed, distorting the local morphology.

The first step is to assess the position and anatomical relations of the carotid bifurcation, which is the inferior limit of this protocol. If present, the non-affected side should be scanned first. With the probe focused on the transverse plane, the operator must identify the important structures of the regions as the sternocleidomastoid muscle (SCM), the internal and external carotid arteries, and the jugular vein. Both the vagus and the spinal accessory cranial nerves may not be visible on the examination (Fig. 1).

The scanning continues up focusing on the external carotid and its branches, heading to the submandibular area. On the submandibular area and still, on the transverse plane the operator should observe the platysma muscle, the relation between the superficial and deep neck fascia and the adjacent tissues, and the tissue pattern focused on inflammation and swelling (Fig. 1).

Second Step: Submental, Sublingual, and Anterior Neck Spaces

**Figure 2:**
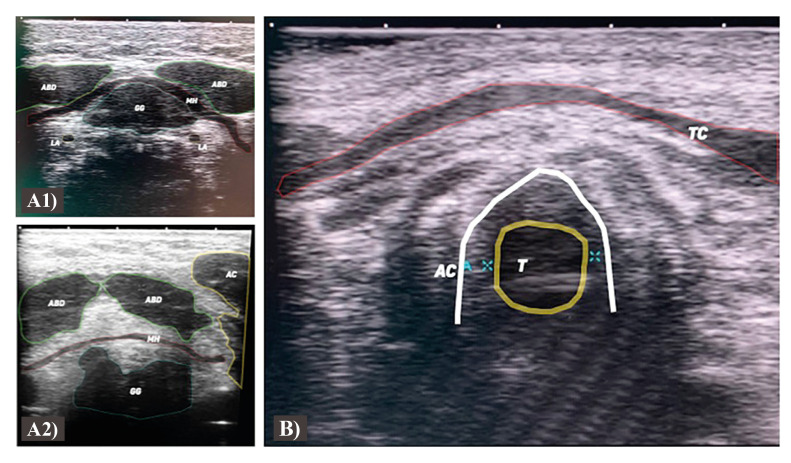
A) Ultrasonography image of the sublingual and mylohyoid region revealing the anterior belly of the digastric muscle, mylohyoid muscle, central raphe, genioglossus muscle, and lingual arteries. B) Ultrasonography image of the central cervical area revealing the thyroid cartilage, airway column, and tube placement.

Further, the operator should focus the probe on the submental and sublingual region, focusing on the structures present in the region as the anterior belly of the digastric muscle, mylohyoid muscle, and geniohyoid muscle, genioglossus muscle, sublingual gland, and both sides lingual artery (Fig. 2). The position of the central raphe of the mylohyoid is marked on the US device, together with the relation between it and the structures of the mouth floor.

Following, the anterior cervical region is scanned focusing on the thyroid, midline, airway column, supra and infrahyoid muscles, larynx, and tracheal rings (Fig. 2).

Step Three: Upper Airway Patency and Volume Assessment

**Figure 3:**
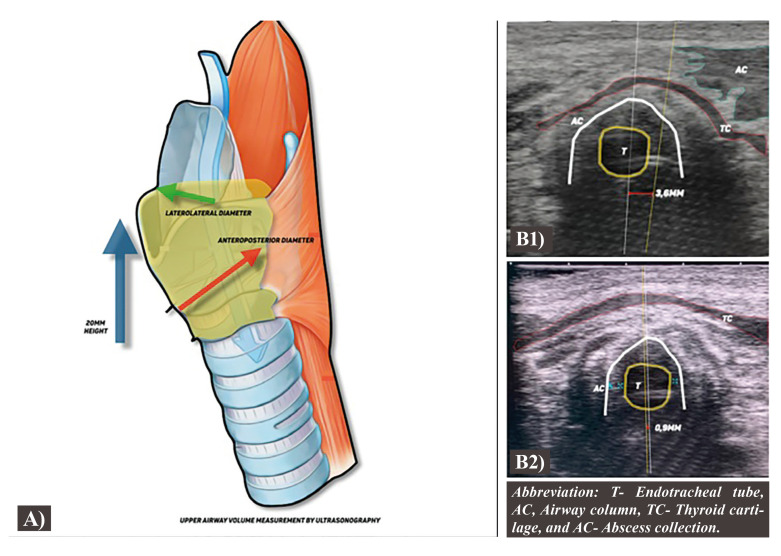
A) Representation of the central neck and upper airway structures, highlighting the area in which the anteroposterior diameter, red arrow, and the laterolateral diameter, green arrow, were measured, at a 20mm height. B) Ultrasonography image of the central cervical area, coming to the airway column deviation by the central raphe of the mylohyoid, not shown on the image and the midline of the airway column. B1- Airway deviation of 3,6mm on the first day of hospitalization. B2- Airway deviation of 0,9mm on the extubation day.

The position of the airway column and the midline are marked. The anteroposterior and lateral diameters of the airway column are measured, and together with that a twenty-millimeter inferior and superior portion of the airway column is examined (Fig. 3).

The upper airway midline was determined by the difference between the central raphe of the mylohyoid and the airway column midline on the central cervical area. The volume of the airway column was obtained using a formula to determine the volume of a cone with a twenty millimeters height (Fig. 3).

- Step Four: Lateral Pharyngeal Spaces

**Figure 4:**
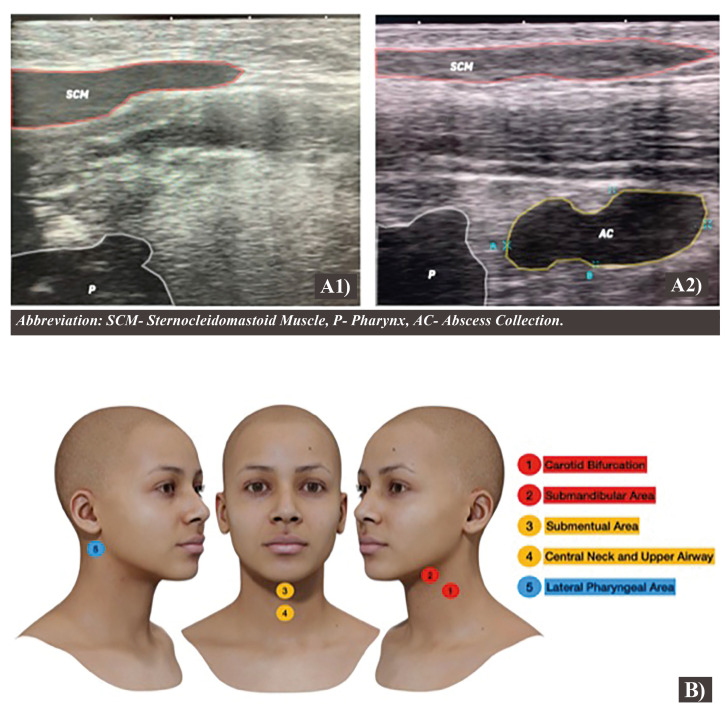
A) Ultrasonography image of the lateral pharyngeal area, left side normal and right side affected. The sternocleidomastoid is the superficial point of this examination, and the pharynx is observed via the airway column shadow. On the affected side, the abscess collection deviates from the airway column and affects the local anatomy. B) 3D-Reproduction of the protocol, illustrating the areas that should be examined.

The lateral pharyngeal area requires a different plane of observation, and the sagittal is the elected one. The probe should be postponed posterior to the mandibular ramus, below the ear lobule and both the gain and the zoom should be placed on more than 5cm for the best exposure. The pharyngeal area is observed by the shadow of the airway column and the SCM is observed on the superficial of the field of view. Collections and deviations of the pharynx could be observed on the examination (Fig. 4).

After the identification of the non-affected side, the examination should be performed on the affected one, focused on the altered anatomical findings. Collections should be visualized and measured in all the planes, if possible.

The relationship between vascular structures and adjacent ones should be assessed by the doppler examination. When related, the distance and the best approach should be documented as well.

The protocol sequence is presented as a 3D -reproduction in Fig. 4.

- Ultrasound-guided drainage

The drainage could be guided by US imaging. With the collection positioned in the center of the image, a large fourteen-gauge (14G) needle is positioned perpendicular to the plane and inserted slowly. A hyperechoic image is observed and the needle is directed to the center of the collection. When positioned, the syringe is aspirated, and the contents are directed to a culture vault and sent for antibiogram examination.

- Data collection methods

The data that were extracted and observed in this study were the medical records of patients, immaginological exams, information from the surgical procedure, and bacterial growth with isolation of the organism. Also, the measures obtained by the ultrasound for anteroposterior diameter, laterolateral diameter, airway volume, midline deviation, and other data such as days of hospitalization, dysphagia, voice change, raised floor of the mouth, dyspnea, and neck swelling, are all red-flags signs described on the literature (6).

- Data analysis

All the data were collected prospectively and tabulated using Numbers ( Apple Inc, Cupertino, CA, USA) and the results were analyzed using the IBM SPSS 25 ( IBM, Armonk, New York, USA). Mean, standard deviation, and percentages were presented as descriptive statistics. Kolmogorov- Smirnov test was performed to evaluate the adhesion of the sample to the normal distribution of the variables. The Two-Tailed Pearson correlation test was used to relate the variables. The degree of statistical significance was considered *p*< 0,05 in a 95% confidence interval.

## Results

In the Present Study, a total of twenty patients met the inclusion criteria (13M, 7F) and were enrolled in this cohort. The results were divided into five parts: ultrasound measures, immaginological findings, US-guided drainage, bacterial profile, clinical features, and statistical correlations.

- Part I: Ultrasound Measures

The anteroposterior and laterolateral diameters were measured, together with airway column volume and midline deviation. The mean value for the anteroposterior diameter was 13,13mm ( ±2,00mm/ ranging from 10,19mm to 16,87mm), in addition, the mean value for the laterolateral diameter was 19,12mm (± 3,77mm/ ranging from 13,60mm to 26,99mm). The airway column volume was obtained as described in the methods and the mean value was 595,39 mm3 ( ± 236,74mm3/ ranging from 290,38mm3 to 1143,68mm3) (Table 1).

- Part II: Immaginological Findings

All the patients enrolled in this study received both X-rays, CT SCAN, and the US for the assessment of the Odontogenic Infection. The X-Ray was the least sensitive exam for the diagnosis of collections, being enough to confirm the condition in only 20% of the patients. The CT SCAN promoted an increase in the sensitivity of the diagnosis for 95%, in addition to that, the US was considered sufficient in 100% of the patients (Table 2).

**Table 1:**
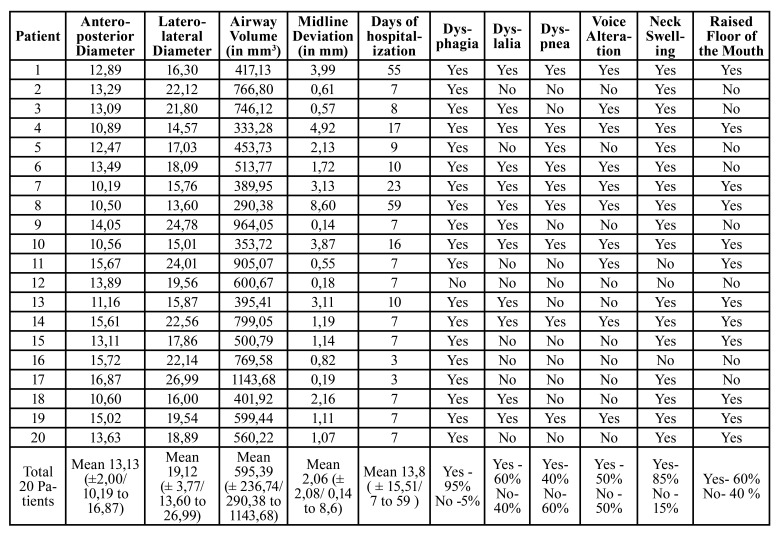
Descriptive results of the study, ultrasound measures, and clinical features.

**Table 2:**
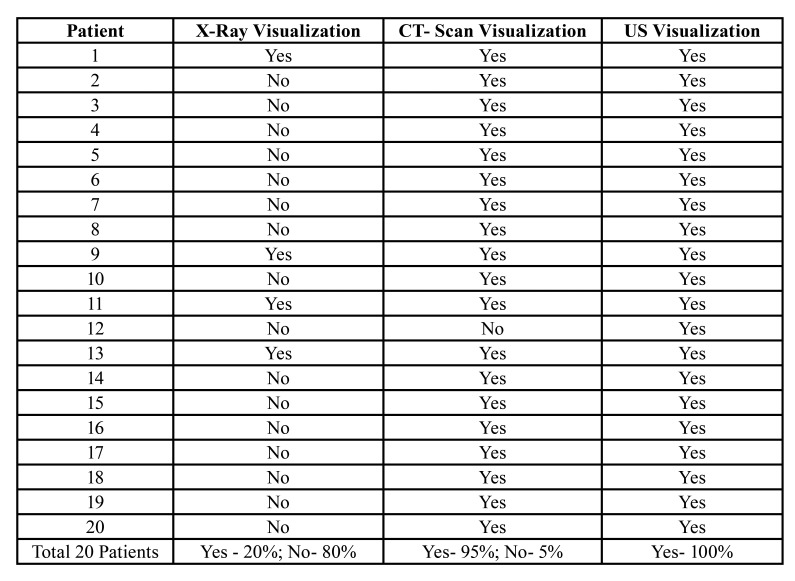
Immaginological findings and comparison between X-Ray, CT- Scan, and US.

- Part III: The US-guided drainage and Bacterial profile

The drainage guided by the US was performed and possible to obtain in 45% of the patients, all the other patients received non-guided drainage, together with that 66% of that amount was possible to isolate the bacteria and profile the population (Table 3).

- Part IV: Clinical Features

The mean time of hospitalization was 13,8 days ( ± 15,51days/ ranging from 7 to 59 days). The clinical red flag signs were evaluated as well and 95% of the patients presented dysphagia, followed by neck swelling (85%), dyslalia (60%), the elevation of the floor of the mouth (60%), voice alteration (50%) and dyspnea (40%) (Table 1).

- Part V: Statistical Analysis and Correlations

The sample was determined as a non-parametric sample, rejecting the normality hypothesis ( *p*>0,05). The correlations between the ultrasound findings and clinical features were performed and the main results are presented in Table 4. The decreased airway volume presented a direct correlation with increased length of stay (*p*= 0,019), increased chance for dyslalia (*p*=0,069), and floor of the mouth elevation (*p*=0,016). In addition to that, increased values of midline deviation are considered risk factor for dysphagia (*p*=0,028), dyslalia (*p*=0,005), dyspnea (*p*=0,001) and elevation of the floor of the mouth (*p*=0,028). The correlations between CT SCAN found no statistical basis ( *P*>0,005). Ultrasound-guided drainage promoted a statistical correlation between bacterial profile and isolation (*p*=0,001).

**Table 3:**
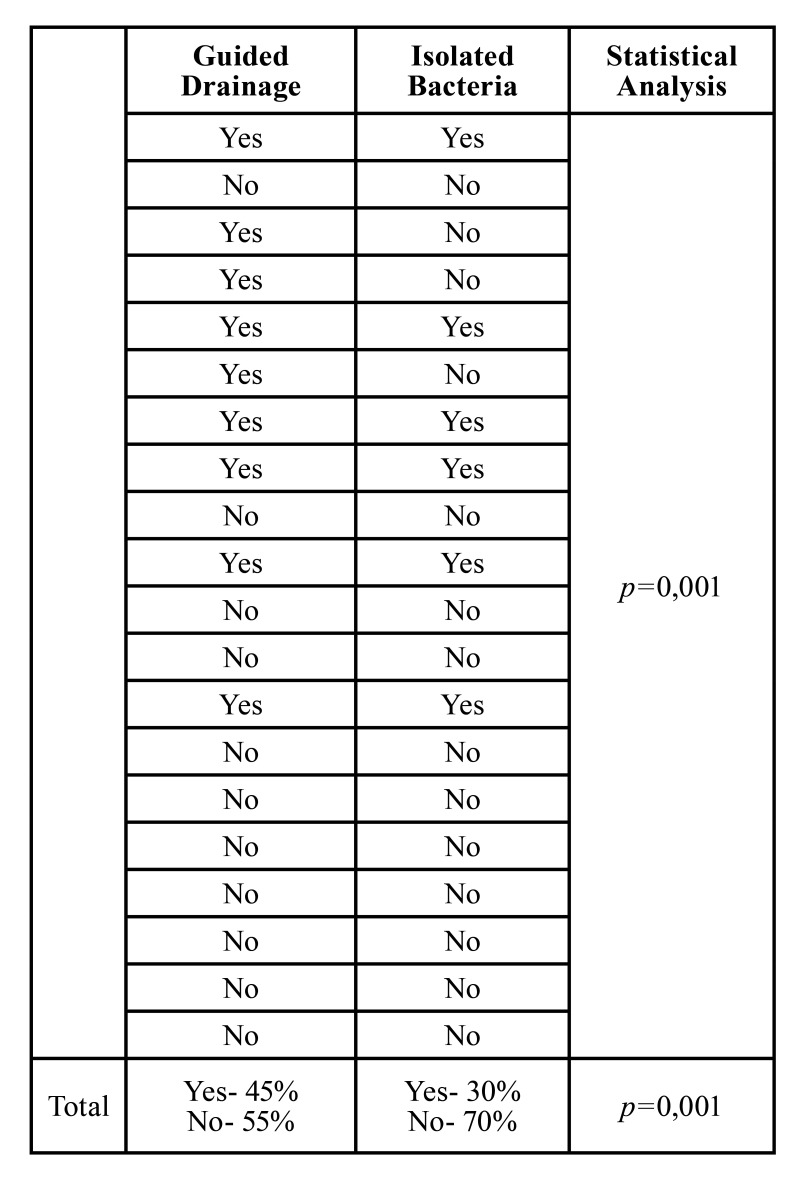
Analysis of US-guided drainage and bacterial isolation.

**Table 4:**
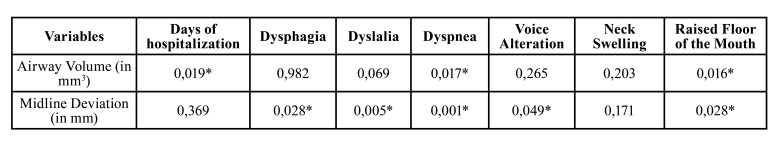
Statistical correlation between the POCUS findings and the clinical features.

## Discussion

The purpose of this study was to validate a protocol for the application of ultrasonography in the assessment, diagnosis, and management of patients with severe odontogenic infection, especially with deep neck involvement. This study confirms the statement that the US is a reliable option with sensitivity comparable to the Gold-Standard CT SCAN. Being directly related to better findings in bacteriological examination and antibiogram. Together with that, the reliability of the ultrasound examination proposed by this protocol for the assessment of the upper airway confirms that the examination could be correlated to the clinical findings.

Previous studies described the indications for the airway POCUS as pre-anesthesia airway evaluation, tracheostomy evaluation, confirmation of the endotracheal tube placement, prediction of the endotracheal tube size, and morphological alteration of the vocal cords (11,12).

Several studies present the US as an important option for imaging the upper neck and submandibular area, especially for pathological findings (13,14). Sethia *et al*., 2017 described the importance and reliability of the US examination on superficial infections of the face region, together with the head and neck. Their study found an elevated sensitivity comparable to the CT SCAN (15). The sensitivity of the US on the diagnosis in the literature range from 33% to 88% (6,15), on this study the sensitivity was 100%, probably due to the location of the collections and the experience of the operator.

The CT SCAN and the MRI, which are still considered the gold standard for the immaginological examination of these patients (1,3,11) are costly and hardly accessible in some hospitals. The POCUS should be repeated during the post-operative time, allowing early extubation and reduced hospitalization time (9).

The POCUS presents a clear advantage as it can be used at bedside evaluation, allowing the best care and assessment of severe and unsTable patients (9), together with that, the findings of this study allow us to address that the diagnosed features observed via POCUS could predict the increase of the hospitalization time ( *p*= 0,019). This study presented an interesting correlation between the airway volume and prolonged length of hospital stay, on the other hand, the airway deviation was not statistically significant, this could be due to the local swelling and also due to the fact that the airways could be crushed but not deviated.

This study collected enough data to address the indication of the POCUS on the upper-airway evaluation by the means of the proposed protocol, as the main findings of the examinations presented a correlation with the red-flag signs for severe odontogenic infections, observed during the physical examination. All the red-flags signs and symptoms, described by Weyh (6), dysphagia, dyslalia, dyspnea, voice alteration, and floor of the mouth elevation presented statistical relevance for either airway column volume alteration or midline deviation. The neck swelling was not related to the POCUS findings for altered upper airways, these could be explained by a large number of factors associated with neck swelling besides deep neck propagation, or by the patient's early search for treatment.

Although the US prevents radiation exposure, there are some counterpoints to this technique as the training process, requires anatomical knowledge of the oral cavity and superficial and deep neck space imaging, however, as described by many previous studies, the training process does not take a long time. Other limitations are user dependence and the limited ability to penetrate bone or air-filled structures (15).

## Conclusions

POCUS is a useful tool, with many advantages for imaging the region in patients with severe odontogenic infection with deep-neck propagation. This protocol is a reliable tool for the diagnosis and management of patients with odontogenic infections, with deep neck involvement, even in an emergency and in unsTable patients. The US guided-drainage promotes a better overview of the drainable collection, collaborating for the correct diagnosis of the bacteriological profile, which reduces the use of empirical antibiotics and promotes the use of the correct therapeutical medication, with undeniable advantages for the patient.

## References

[1] Taub D, Yampolsky A, Diecidue R, Gold L (2017). Controversies in the management of oral and maxillofacial infections. Oral Maxillofacial Surg Clin North Am.

[2] Tapiovara L, Back L, Aro K (2017). Comparison of intubation and tracheostomy in patients with deep neck infections. Eur Arch Otorhinolaryngol.

[3] Arias-Chamorro B, Contreras-Morillo M, Acosta-Moyano A, Ruiz-Delgado F, Bermudo-Añino L, Valiente-Álvarez A (2011). Multiple odontogenic abscesses. Thoracic and abdomino-perineal extension in an immuno competent patient. Med Oral Patol Oral Cir Bucal.

[4] Reynolds SC, Chow AW (2007). Life-threatening infections of the peripharyngeal and deep fascial spaces of the head and neck. Infect Dis Clin North Am.

[5] Moghimi M, Baart Ja, Karagozoglu H, Farouzanfar T (2013). Spread of odontogenic infections: A retrospective analysis and review of the literature. Quintessence Int.

[6] Weyh AM, Dolan JM, Busby EM, Smith SE, Parsons ME, Norse AB (2021). Validated image ordering guidelines for odontogenic infections. Int J Oral Maxillofac Surg.

[7] Lawrence R, Bateman N (2017). Controversies in the management of deep neck space infection in children: an evidence-based review. Clin Otolaryngol.

[8] Plaza Mayor G, Martinez-San Millan J, Martinez-Vidal A (2001). Is conservative treatment of deep neck space infections appropriate?. Head Neck.

[9] Costa SM, Lanes Silveira R, Amaral MBF (2021). Ultrasonography on the Early Postoperative Control of Severe Odontogenic Infections. J Oral Maxillofac Surg.

[10] Even-Tov E, Koifman I, Rozentsvaig V, Livshits L, Gilbey P (2017). Pre- procedural ultrasonography for tracheostomy in critically ill patients: a prospective study. Isr Med Assoc J.

[11] Shibasaki M, Nakajima Y, Ishii S, Shimizu F, Shime N, Sessler DI (2010). Prediction of pediatric endotracheal tube size by ultrasonography. Anesthesiology.

[12] Hardee PS, Ng SY, Cashman M (2003). Ultrasound imaging in the preoperative estimation of the size of tracheostomy tube required in specialized operations in children. Br J Oral Maxillofac Surg.

[13] Dogan S, Ekinci A, Demiraslan H (2016). Ultrasonography and contrast-enhanced CT findings of tularemia in the neck. Diagn Interv Radiol.

[14] Choi HI, Choi YH, Cheon JE, Kim WS, Kim IO (2018). Ultrasonographic features differentiating thyroglossal duct cysts from dermoid cysts. Ultrasonography.

[15] Sethia R, Mahida JB, Subbarayan RA, Deans KJ, Minneci PC, Elmaraghy CA (2017). Evaluation of an imaging protocol using ultrasound as the primary diagnostic modality in pediatric patients with superficial soft tissue infections of the face and neck. Int J Pediatr Otorhinolaryngol.

